# Does China have a public debate on genetically modified organisms? A discourse network analysis of public debate on Weibo

**DOI:** 10.1177/09636625211070150

**Published:** 2022-01-27

**Authors:** Yan Jin, Simon Schaub, Jale Tosun, Justus Wesseler

**Affiliations:** Wageningen University, The Netherlands; Teagasc—Irish Agriculture and Food Development Authority, Ireland; Heidelberg University, Germany; Wageningen University, The Netherlands

**Keywords:** China, discourse network analysis, genetically modified organisms (GMOs), public debate, Weibo

## Abstract

We examine stakeholder participation in the online debate on genetically modified organisms in China and assess how the debate has changed over time. Therefore, we compare messages posted between 2013 and 2020 on the Chinese microblog website Weibo by using discourse network analysis. Our findings reveal strong opposition to genetically modified crops, along with the existence of two competing coalitions of supporters and opponents. We further observe an increasing number of posts supporting genetically modified organisms by the public in recent years. Consequently, there is an indication that the positions of stakeholders have changed over time. We discuss the policy implications for China and draw conclusions for other countries.

## 1. Introduction

China is pursuing an active biotechnology strategy. In 2010, research funding for genetically modified organisms (GMOs) amounted to around 2.5 billion US dollars, having increased at a rate of 22% per year since 2005 (Cai et al., 2016). In 2008, the Chinese government announced a GMO project to support research and development for new genetically modified (GM) crop varieties through 2020, with a total budget of 35 billion US dollars (Cai et al., 2016). Published in 2016, the *13th Five-Year Plan for Science and Technology Innovation* officially promotes the development of domestic GM crops and their release into the environment (State Council, 2016). Chinese farmers were also among the first farmers cultivating GM crops, virus-resistant tobacco in 1988 (Thomson, 2021).

Despite strong political support, the cultivation of GM crops is actually prohibited in China, with the exception of GM cotton and papayas (Wong and Chan, 2016). At first glance, the government’s response to GM crops thus seems contradictory. Meanwhile, a lack of public confidence in GM crops due to historical events (see “The initiation of the debate on GM crops in China” section) has sparked a heated online debate as GM crops approach commercialization. This debate exhibits similarities to those observed in the European Union and the United States (Castellari et al., 2018; Tosun and Schaub, 2017). At first glance, it might not seem likely that the Chinese government would consider public opinion when making policy decisions, as governmental actors in China do not depend on competitive public elections to remain in office (Acemoglu and Robinson, 2012; Easterly, 2013). In this regard, the government’s current policy of prohibiting the cultivation of GM crops might appear surprising, possibly implying that the government does indeed consider public opinion, at least to some extent. Furthermore, previous research has demonstrated that the use of mass media can influence public opinion and steer the public debate (Rim et al., 2020), which subsequently affects the level of GM food policy in a country (Herring and Paarlberg, 2016). Greenpeace, which opposes GMOs, has become an important player in China since the early 2000s supported by the rise of social media (Thomson, 2021).

In this study, we examine the public debate on the use of green biotechnology and, in particular, the cultivation and use of GM crops. To this end, we investigate underlying lines of conflict related to the authorization of GM crops in China. We are particularly interested in identifying distinct coalitions of actors^1^ holding opposing views on GM in the policy debate, as well as the composition of such coalitions and possible changes in their membership in the past 7 years.

Research shows that there exist regional differences in public opinion; for example, in some regions, opinions could vary in conservatism due to sociocultural differences (Weakliem and Biggert, 1999). Due to the heterogeneity of natural resources or information resources, regional differences also present challenges with regards to policy implementation (Colley and Brown, 2015) and an efficient allocation of goods (Liu et al., 2020). Therefore, regional differences may also materialize in the stakeholder participation in online public debates.

In the European Union, two stable actor coalitions either supporting or opposing GMOs have been observed (Tosun and Schaub, 2017). Coalitions play a role in public debates in many other countries as well (Herring and Paarlberg, 2016). We analyze the public debate based on a network analysis of posts placed on Weibo, the most popular social media platform in China with more than 203 million daily users (Business Statistics, 2020). More specifically, we analyze the dynamics of Weibo debates on GM crops from the earliest entries we could trace until the end of our observation period, that is, March 2013–April 2020. To the best of our knowledge, this is the first study to focus on the online debate concerning GMOs in China. Based on Weibo posts, our results reveal strong opposition to the cultivation of GM crops in China. The Weibo debate was characterized by two competing advocacy coalitions, as well as by regional differences and changes in coalition membership over time.

## 2. Empirical background of the analysis

In this section, we provide background information on the cultivation and regulation of GMOs in China to set the stage for the subsequent analysis.

### GMOs in China

The contribution of biotechnology to crop productivity has been debated among scientists, as well as in other societal groups (Huang et al., 2004; Rotolo et al., 2015). Studies have noted the advantages of adopting GM crops, including increased crop productivity, associated environmental benefits, and improved nutrition (Barrows et al., 2014; Bennett et al., 2013). Others mention the potential risks (Myers et al., 2016; Van Acker et al., 2017), including the increasing resistance of insect pests and unknown interaction between residues of glyphosate-based herbicides with GM cultivars. Yet, others argue that other strategies based on agroecological concepts could also achieve the intended objectives of GM crops without exposing society to the potential risks (Maghari and Ardekani, 2011).

Despite the existing controversy, GM crops accounted for around 192 million hectares in 2018. The five leading countries in terms of GM crop cultivation are Argentina, India, the United States, Brazil, and Canada. These countries account for 91% of GM crop acreage worldwide (ISAAA, 2018). By 2018, 70 countries had adopted GM crops, with 26 countries planting and 44 countries importing such crops (ISAAA, 2018). China approved the commercialization and cultivation of GM cotton in 1997, and GM papayas in 2006 (Wong and Chan, 2016). In 2017, GM cotton accounted for 2.78 million hectares, with GM papayas covering 2.8 million hectares in China (ISAAA, 2017). Since 2006, no additional GM crops have been approved for cultivation in China. Although several varieties of GM crops have undergone strict risk assessment and a complex regulatory process with regard to cultivation, the final approval is pending (Jin et al., 2019b).

Since 2000, China has pursued an active biotechnology strategy, with substantial investment in research and development for GMOs. Despite a cut in funding for research on GMOs due to public resistance in the early 2010s, the government resumed its support for research and commercialization with regard to GMOs in 2016. The announcement of the *13th Five-Year Plan for Science and Technology Innovation* for promoting the development and application of domestic GM crops in July 2016 represented a milestone. The report released by the Ministry of Agriculture and Rural Affairs (MARA) on increasing public biotechnology literacy to advance the commercialization of GM crops in July 2018 constituted a second milestone. The strategy includes closer cooperation between local governments and the media (MARA, 2018), including Weibo, for increasing the public’s literacy concerning biotechnology.

### The initiation of the debate on GM crops in China

GM crops were introduced to the public in a “negative” way, with three critical events that had a corresponding impact on public opinion.

The first event involved the issuance of biosafety certificates for insect-resistant GM rice on October 22, 2009. The biosafety certificates for GM rice indicated that it was considered as safe as conventional rice, for both humans and the environment, and that it was thus ready for commercialization (Jin et al., 2019a). At first, MARA did not disclose any information on the results of the health and environmental studies, even though GM rice had successfully passed various laboratory tests, in addition to three phases of field trials testing potential environmental risks. It would not be until 5 years later when the certificates were set to expire and renewal was requested, that MARA would disclose the results (Cao, 2018). In addition, the public did not know the identity of the members of the biosafety committee, who had decided to issue the biosafety certificates for GM rice, nor were the biosafety criteria generally known. As a result, people became increasingly critical of the transparency of the process on social media (Cao, 2018).

GM rice had already been cultivated illegally before its official approval (Zi, 2005). In February 2005, Greenpeace collected samples of rice from various sectors in the supply chain in Hubei province, where GM rice had initially been developed. Greenpeace sent the samples to GeneScan (a GMO laboratory in Germany) for testing. Of the 25 samples, 19 were found to include GM rice, which should have still been in the experimental stage (BBC, 2005; Cao, 2018). Greenpeace revealed similar information on the illegal cultivation of GM rice again in May 2014, after having tested rice bought at random in supermarkets in the province of Hubei. Subsequently, MARA and the Chinese government were blamed online for the weak implementation of laws and regulations on GMOs.

The third event alludes to “golden rice,” a type of GM rice that developers genetically enriched with beta-carotene, with the objective of producing a fortified food to be grown and consumed in areas with a shortage of dietary vitamin A (Black et al., 2008). In 2012, a nutrition test on *golden rice* was performed in the province of Hunan. The study was funded by the US National Institute of Diabetes and Digestive and Kidney Diseases, and the US Department of Agriculture (Hvistendahl and Enserink, 2012). The research team included four US-based and three China-based researchers. The researchers have been accused of not properly informing the study participants about the origin of the Vitamin A enhanced rice (Dubock, 2014) by referring to the rice as beta-carotene-containing instead of GM rice. The implementation and publication of the research resulted in disputes among the scientists involved and authorities supervising and allocating grants for research. The three China-based researchers were ultimately removed from their positions, and financial compensation was granted to those subjects involved in the nutritional study (Hvistendahl and Enserink, 2012). The incident nevertheless caused substantial damage to public trust in science, causing further deterioration in the Chinese GMO research environment as a whole (Dubock, 2014; Qiu, 2012).

The events and reactions of the government, which had been perceived as not taking the public interest into consideration, decreased public confidence in GM crops, as well as trust in MARA and the government (Chow, 2019; Cui and Shoemaker, 2018; Pray et al., 2018). In both 2016 and 2018, government documents established official support for GMOs (MARA, 2018; State Council, 2016). The loss of confidence and trust, together with government support for GMOs, generated a tense atmosphere for GM crops in China, as well as a heated debate on social media (Cao, 2018).

## 3. Theoretical considerations on the Chinese policy response to GMOs

In democratic political systems, governmental actors are dependent on competitive elections to remain in office. They consequently tend to be responsive to public opinion when making policy decisions (Soroka and Wlezien, 2009; Wlezien, 2017). In autocracies (e.g. China), this mechanism does not operate directly, due to the lack of competitive elections. According to recent literature, however, some authoritarian regimes are also responsive to societal actors, albeit for different reasons (Kornreich, 2019; Popović, 2020; Tang et al., 2018). Central and local governments have increasingly used public consultation to gather information on public opinion to prevent potentially threatening collective actions that could destabilize the regime (Jiang et al., 2019; Kornreich, 2019; Li et al., 2016). Controlling the public debate is one way in which the Chinese government has tried to influence public opinion. Traditionally, the regime has applied various censorship methods to suppress online criticism of the regime (Chang and Lin, 2020; Keremoğlu and Weidmann, 2020; King et al., 2013). In addition, the government has participated in the public debate, albeit somewhat indirectly, by hiring Internet commentators to take part in online discussions anonymously spreading government-friendly views (Han, 2015; King et al., 2017).

Plans by the Chinese government to establish large-scale cultivation of GM crops have faced increasing public opposition (Cui and Shoemaker, 2018). Based on a nationwide Chinese consumer study, the main reason for opposition is that “GM food may have unknown risk to human beings, such as some genetic defects, which may affect human beings for many generations. It will take a long time to validate the safety of GM food using scientific experiments” (Cui and Shoemaker, 2018). Negative public opinion on the commercialization of GM crops and associated fears of diminishing regime stability could potentially explain why the government has been hesitant to lift the ban on GM crop cultivation. Instead of revoking its overall plan on GMO commercialization, however, the government has cemented its intentions to foster GM crop cultivation as mentioned above. The Chinese government is thus likely to try to influence public opinion in ways that will allow the implementation of its biotechnology plans without the risk of losing public support. As explained above, one way that the government could try to change public opinion would be to intervene in the public debate.

In line with existing research on GMOs (e.g. Tosun and Schaub, 2017), we expect to observe a controversial debate between supporters and opponents of GMO commercialization since the issue tends to polarize. We expect to observe efforts from the Chinese government to influence public opinion on GMO commercialization for two reasons: first, given the autocratic features of the regime, it can influence the public debate both directly and indirectly by changing the behavior of other participants in the debate (Han, 2015); second, even in autocratic systems, public opinion matters and existing research has shown that central and local governments in China do indeed respond to it (Li et al., 2016).

We find evidence of controversial debate in the literature that seems also to have taken place in the case of GMOs in China (Cao, 2018). With the expected opposition toward its plan to intensify the application of modern biotechnology in agriculture, we expect the government would aim to implement the biotechnology plan while attempting to ensure public support by also influencing the debate on social media. Its responsiveness to public opinion is largely due to concerns about political stability, loss of regime legitimacy, and the threat of rebellion (Chen et al., 2016; Popović, 2020). Hence, we expect to observe changes in the debate and the behavior of various types of actors in response to crucial policy events, like the release of the current *Five-Year Plan* in 2016. This expectation is motivated by research in communication research on extreme and nonextreme events (Stieglitz et al., 2018). We further expect to observe differing behavior from the central government and local governments since public dissatisfaction poses a threat to the career prospects of lower-level officials, and therefore, they are more strongly motivated to prevent any unfavorable public opinion (Chen et al., 2016; Li et al., 2016).

## 4. Methodology used for the analysis of the public debate on GMOs

Discourse network analysis is a versatile methodological approach that is widely used to describe the structure of policy debates, the presence of coalitions, and the dynamics of policy debates over time (Leifeld, 2017, 2020) It has been applied in a variety of policy sectors (Breindl, 2013; Fisher et al., 2013; Rinscheid, 2015). Discourse networks capture verbal interactions between actors making public statements on a policy issue in a systematic and quantifiable way (Leifeld, 2016, 2017) A discourse network consists of a set of dates, actors, concepts, and actors’ agreement or disagreement on concepts. In this study, discourse network analysis is used to trace the online debate on GM crops in China, as well as to visualize competing advocacy coalitions, scrutinize their formation, and analyze regional differences and time dynamics to investigate the formation of actor coalitions among various types of stakeholders and how such coalitions contribute to the policy debate. We also include locations in the discourse network to indicate the regional distribution of the GMO debate. The information is coded using Discourse Network Analyzer software based on statements posted by actors (Leifeld et al., 2019).

To study the formation of the advocacy coalitions, we adopt a one-mode actor congruence network, which captures the similarity of actors based on whether they agree or disagree with certain concepts associated with GM crops. More specifically, positioning on these concepts indicates whether actors support or oppose GM crops. Using a congruence network approach then enables us to analyze how similar actors are in their positioning. The resulting congruence network matrix contains actors in rows and columns, with cell values indicating the similarity of actors. The more often two actors co-support or co-oppose a concept, the higher their similarity. We use the *Jaccard similarity measure*^2^ to normalize the network matrix, thereby correcting potential biases produced by highly active nodes (Leifeld, 2017). Values within the normalized network matrix range between 0 and 1 with higher values indicating a higher similarity of two actors in their positions on GM crops. We use the *Fruchterman–Reingold force-directed placement algorithm* provided by the *ggnet2* function within the *R* software (R Core Team, 2020) to visualize the network with *network graphs*, where actors as *nodes* are linked by *ties* if they co-reference at least one concept. The algorithm is commonly applied in social network analysis and places groups of nodes, characterized by higher similarity, closer together (Fruchterman and Reingold, 1991). Thus, actors with higher degrees of similarity (i.e. more co-referenced concepts) are positioned closer to each other in the graph. The absolute distance and the length of ties between actors cannot be meaningfully interpreted.

We use social network cluster analysis to identify communities. In social networks, clustering involves grouping nodes into clusters based on similarity, with “communities” of nodes sharing common properties and characteristics. Identifying communities helps to explain different patterns of supporting or opposing arguments. The analysis we perform is based on the *Walktrap algorithm*, which is suitable for graph patterns with a strong community structure (Charrad, 2016).

Pons and Latapy (2006) developed the *Walktrap algorithm* to identify communities in networks via random walks. Random walks mean the algorithm moves from one node to another through a random choice at each step. The *Walktrap algorithm* is a hierarchical clustering algorithm to search for densely connected subgraphs through random walks, in addition to identifying the optimal number of clusters (Charrad, 2016). Starting from a totally nonclustered partition, the distances between all adjacent nodes are computed. The two closest communities are chosen and are merged into a new one; the distances between communities are then updated based on random walks (Yang et al., 2016). Short-distance random walks tend to stay in the same community. Detailed mathematical descriptions can be found at Pons and Latapy (2006).

We use the *walktrap.community function* within the R software (R Core Team, 2020) to analyze changes in community formation over time. Within the network graphs, nodes are colored to indicate different actor types and are shaped differently according to actors’ identified community membership.

## 5. Data used in the analysis

The social media platform Weibo is the data source from which we collected statements from various actors. This Chinese microblogging website (similar to Twitter in Western countries) has been one of the most popular and widely used platforms in China since 2009. The number of monthly active users of Weibo reached 486 million in 2019 (Weibo, 2019). The most frequently used feature of Weibo is the ability to follow hot topics and news (Business Statistics, 2020). Due to a lack of censorship compared with other mass media in China, Weibo plays an important role in disseminating information, sharing various political viewpoints, interacting with political campaigns, and having online debates about controversies (Su, 2019). Social interactions on Weibo have built various kinds of public spheres on Weibo with shared characteristics, such as critical consciousness and democratization of public opinion (Rauchfleisch and Schäfer, 2015; Su, 2019). Given the influence of mass media on public opinion (Delshad and Raymond, 2013; Rim et al., 2020), we assume that Weibo discussions about GM crops may influence perceptions and opinions on the topic.

According to a 2016 survey, most people in China obtain their information on GMOs through the Internet (Cui and Shoemaker, 2018). Research has shown that actors who strategically engage in participation (e.g. opinion leaders) influence Weibo users’ perceptions (Su, 2019). We can therefore assume that actors interested in influencing the public engage in Weibo discussions (Hestres, 2014).

Weibo contains a massive amount of information about GM crops. Most statements are descriptive (e.g. news about the development of GM crops), however, and have already been analyzed in a previous study focusing on semantic networks and the frequency of keywords about GMOs (Li et al., 2019). Xu et al. (2020) investigate how information veracity and account verification influence the dissemination of GMO-related information on Weibo. To analyze how different stakeholders try to influence the public over time, we focus on statements from various actors with a clear attitude either supporting or opposing GM crops. We selected a special function (i.e. Weibo topics) to draw a sample from the debate in which Weibo users express themselves according to the topics. We relied on the popularity of participation in the debate as indicated by the number of contributions (comments and re-posts) to select the topics. Fourteen debate topics were selected for this study sorted by the number of contributions (see Supplemental Appendix 1). Due to the settings of Weibo, the earliest statement we could trace for the topics covered in our study was from March 2013.

Based on the selected Weibo topics between March 2013 and April 2020, we manually coded 778 statements in which Weibo users expressed a clear attitude toward GM crops without considering neutral statements, that is, clear expression either supporting or opposing GMOs. Within the collected posts, we coded statements on relevant concepts, as derived from a previous study on GMOs in the European Union (Tosun and Schaub, 2017) and adjusted to reflect the situation in China resulting in eight concepts in total. Concept 1 indicates whether actors agreed that transparency concerning GM crops was already existent in China, and Concept 2 concerns their agreement that the current approval process included strict risk assessment and regulations. Concept 3 identifies actors rejecting GMO crops due to long-term uncertainties associated with them. Concept 4 indicates whether actors attributed negative effects on public health, the environment, or traditional agriculture to GMOs. Concept 5 indicates whether actors supported the promotion and cultivation of GM crops, and Concept 6 concerns support for placing GMOs on the market as either food or feed. Concept 7 refers to whether actors point to a lack of knowledge on biotechnology in general as a reason for opposing GM crops. Concept 8 concerns trust in the government with regard to the approval process and the implementation of laws and regulations in general.

## 6. Results and discussion

### Actor type

In Table 1, we provide an overview of 10 types into which we assigned actors and their organizations. Most of the statements (84%) were made by anonymous individuals,^3^ followed by members of the scientific community (e.g. universities and research institutes), governmental entities (e.g. MARA, local and central governments), the business community (e.g. biotechnology and agricultural technology companies), the media (e.g. CCTV and newspapers), foreign governments and legislatures (e.g. the Russian government), international organizations (e.g. the UN Food and Agriculture Organization), international environmental groups (Greenpeace), and a court (the Henan Court). One statement stemmed from the US Consulate in Shenyang; it was categorized as “other.” Although the absolute number of statements from the government may seem small, it is important to note that Chinese government officials seldom engage in online debate directly. Instead, they tend to influence the debates indirectly by asking regular users to advocate the government’s position (Han, 2015)

**Table 1.** Number of statements per actor group.

Actor type	Total	Share in %	Supporter	Opponent
Anonymous	655	84.2	65	590
Science	33	4.2	33	0
Government	32	4.1	21	11
Business	22	2.8	10	12
Media	13	1.7	8	5
Foreign government and legislature	11	1.4	2	9
International organization	8	1	6	2
Environmental group	2	0.3	0	2
Court	1	0.1	0	1
Other	1	0.1	1	0
Sum	778	100	146	632

Source: Data collection from Weibo (2013–2020).

### Regional distribution

It is important to mention regional heterogeneity. People in different regions may have different opinions on GMO cultivation based on their personal experience and access to natural, economic, and information resources. In general, people from the eastern part of China participated more actively in Weibo debates on GM crops than those from the western part and remote inland regions. People from Beijing were the most active (27% of total participation), followed by Hubei (9%), Guangdong (8%), Jiangsu (7%), and Shanghai (5%). Beijing, Shanghai, and Guangdong are regarded as the three most developed regions in China. Consciousness concerning GM crops might therefore be relatively high in these regions, and inhabitants might be eager to express their opinions online to influence policy-makers. In both 2005 and 2014, illegally planted GM rice was found on sale in Hubei (see “The initiation of the debate on GM crops in China” section), possibly explaining the active participation of actors in that province.

### Coalition formation in the public debate

**Figure 1.**
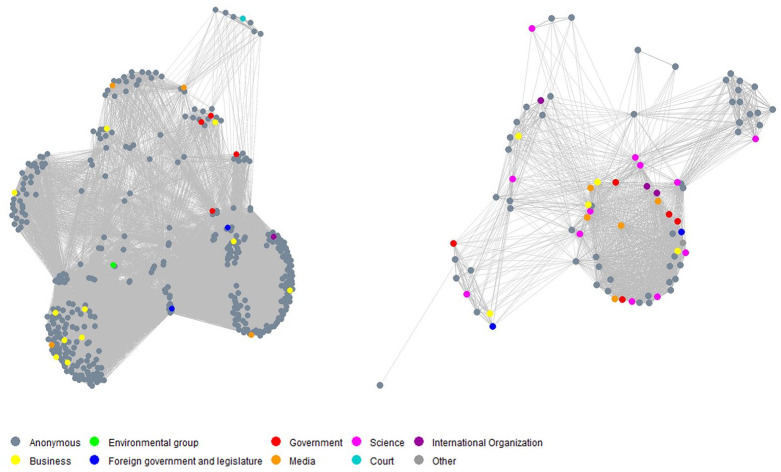
Visualization of the actor congruence network (left: opponents; right: supporters).

The actor congruence network for the full observation period is depicted in Figure 1.^4^ When analyzing the network structure, separate groups of actors in the graph can be interpreted as competing coalitions with higher internal similarity regarding their positions on GM crops (Leifeld, 2016). Based on the congruence network depicted in Figure 1, there are two clearly separable coalitions, each consisting of actors with similar positions toward the concepts.

Actors in the cluster on the left-hand side of Figure 1 form a GM crop opposing coalition whereas actors in the cluster on the right-hand side are part of a supporting coalition. The opponent coalition comprises six times more actors than the supporter coalition. Anonymous individuals are dominant in both competing coalitions. Both coalitions comprise various actor types, which are indicated by different node colors in the network graph. The supporter coalition includes members from the government, international organizations, the business community, media, science, foreign governments, and the legislature, while the opponent coalition includes members from the government, foreign governments, business, media, environmental groups, international organizations, and courts.

Domestic governments are represented in both coalitions, thus indicating conflicting domestic views at different levels. For example, this was the case for Heilongjiang, where the provincial government issued a legislative ban on the cultivation, processing, and sale of GM crops, as well as any agricultural products containing GM ingredients (Heilongjiang Government, 2017). Meanwhile, statements from MARA were posted on Weibo as well, promoting GM crops by citing scientific evidence (e.g. “All GM crops approved for commercialization are safe”). MARA officials in different circumstances stressed similar statements, although they also expressed disappointment (e.g. “Our government has been promoting GM crops for many years. Judging from those rumors online, however, the power of promotion has obviously been insufficient.”)

In our database, all actors identified as scientists were in the supporter coalition, with affiliations with universities and research institutes including Fudan University, Huazhong Agricultural University, China Agricultural University, the Chinese Academy of Science, the Chinese Academy of Engineering, and the Chinese Science Association. These scientists posted statements to support the safety of approved GM crops. Examples included: “China cannot wait for the commercialization of GM crops. The blockage of commercialization is having a negative impact on its research and development as well”; and “We should allow GM rice on the market within five years.”

Members of the business community appeared in both coalitions, representing agricultural technology companies, biotechnology companies, food companies, and companies in other sectors (e.g. media, trade, and culture communication). Statements from companies were quite diverse. For example, supporters posted: “We hope that the public will soon be able to understand GM crops and biotechnology correctly.” “Pesticides smell bad, and they are so bad for farmers’ health. Don’t you think that it is actually necessary to research and develop GM crops?” Members from the business community posted opposing posts, including: “Our company does not use any raw materials that are based on GMOs, and GM food is not suitable for China”; “GM crops are worse than opium.”

The classic media may play a role in influencing perceptions of GMOs on Weibo through various online news platforms (e.g. CCTV, People’s Daily, and ArnetMiner Academic News). Posts included interpretations of research results (e.g. “Research has proved the safety of GM crops as food, consistent with findings released by the food standard agencies in the United States, Canada, and Australia”). Others shared different opinions (e.g. “Commercialization of GM rice might be fatal for traditional rice varieties”; “Illegal cultivation of GM rice . . .Where is the so-called very strict regulation?” and “The labeling of GM products on the market is not clear, which damages consumers’ ‘right to know’”).

As shown in Figure 1, actors of different types appear in both coalitions (except for the actor types Court and Other), possibly due to diverse opinions held by different actors within the same actor type (as discussed above), inconsistency of actors over time, or both.

### Time dynamics in the public debate

We analyzed the public debate over time to determine whether various stakeholders changed their positions. Based on recent government actions concerning GM crops (see “GMOs in China” section), we split the analysis of the public debate into three periods and searched for differences between these periods. Period 1 (May 2013 to July 2016) covers the time before the official announcement of policy support by the government. Period 2 begins in August 2016, after the official announcement of policy support, and ends in July 2018, right before the government started to cooperate with the media in its campaign for GM crops. Period 3 runs from August 2018 (marking the beginning of this cooperation) through April 2020 (the end of our observation period).

**Figure 2.**
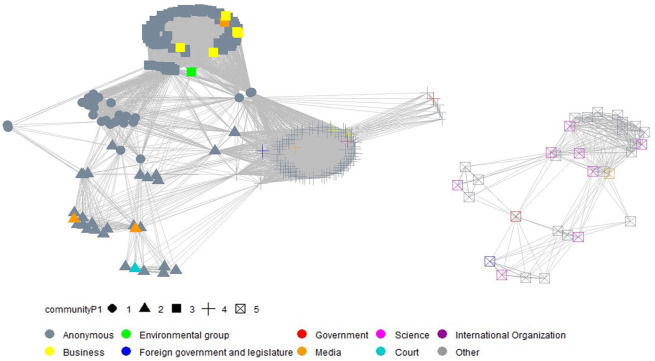
Visualization of time dynamics of the congruence network (left: opponents; right: supporters) and social network cluster analysis, based on the *Walktrap algorithm* (period 1, May 2013 to July 2016, 352 statements).

**Figure 3.**
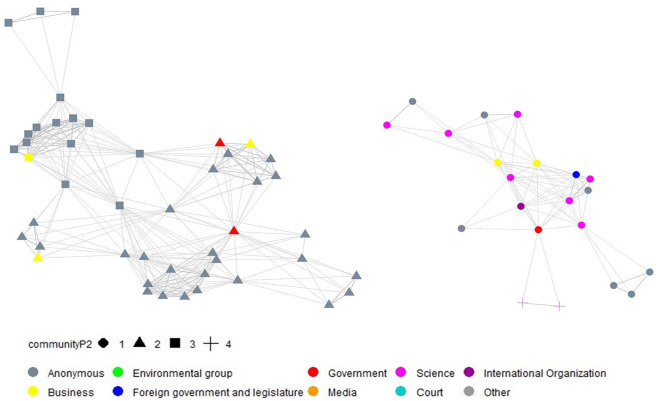
Visualization of time dynamics of the congruence network (left: opponents; right: supporters) and social network cluster analysis, based on the *Walktrap algorithm* (period 2, 2016.08-2018.07, 104 statements).

**Figure 4.**
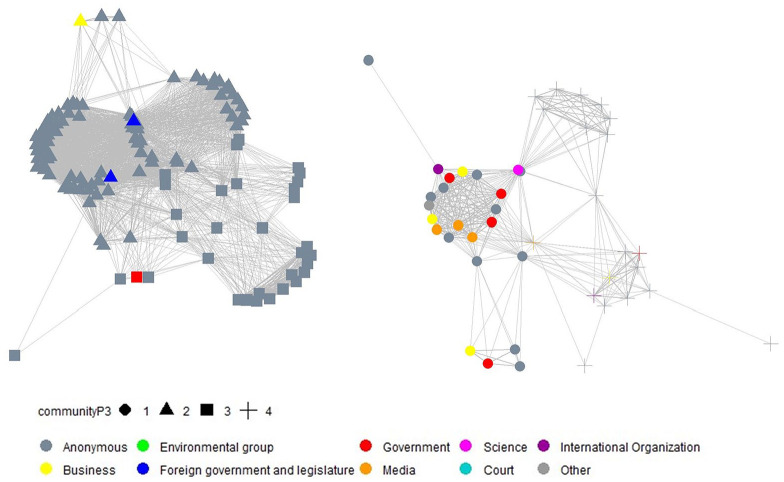
Visualization of time dynamics of the congruence network (left: opponents; right: supporters) and social network cluster analysis, based on the *Walktrap algorithm* (period 3, August 2018 to April 2020, 322 statements).

For each period, we derived a separate congruence network. These networks are visualized through network graphs in Figures 2–4^5^, by using the same approach as applied for Figure 1. In addition, the shape of the nodes in the derived network graphs visualize the results of the cluster analysis.

Similar to our analysis on the whole observation period, we find two competing coalitions in all of the three periods, which indicates a certain stability of the conflict over time. For each period, the coalition on the left represents opponents of GM crops, with supporters depicted in the coalition on the right. The opponents were more active in participating in the Weibo debate in all three periods.

As shown in Figure 2, the actor composition of the opponent coalition in period 1 was more diverse than in subsequent periods. It included members from the business community, the media, environmental groups, governments, courts, and international organizations. The supporter coalition was dominated by scientists, aside from anonymous individuals.

The social network cluster analysis reveals five different clusters within the network: The opponent coalition consists of four clusters whereas the supporter coalition is not further subdivided into separate clusters. Four different kinds of opponents included those concerned primarily about the potential risk of GM crops due to distrust in the government (cluster 1); those doubting the transparency of the current risk assessment and regulations (cluster 2); those believing that GM crops would have negative effects on public health, the environment, or traditional agriculture (cluster 3); and those opposing GM crops, either due to long-term scientific uncertainty or for no specific reason (cluster 4). Cluster 5 includes all actors supporting GM crops for other reasons.

After the government announced its policy support in period 2, media actors and many other actors stopped posting statements on Weibo in opposition to GM crops. The opponent coalition in this period consisted largely of anonymous individuals, along with some actors from local governments and businesses. In the supporter coalition, business actors joined the scientific actors. The social network cluster analysis for period 2 reveals four different communities, two belonging to the supporter coalition and the other two belonging to the opponent coalition. The supporting coalition is now subdivided into two clusters, with one cluster consisting of actors explicitly supporting the promotion and cultivation of GM crops. This cluster nevertheless contains only two statements from the science group: one from the Chinese Academy of Agricultural Sciences and the other from Huazhong Agricultural University. In contrast to the four clusters in the opponent coalition in period 1, the opposing coalition is only characterized by two clusters in period 2. One opposing cluster is small, comprising actors concerned about the negative effects of GMOs on public health, the environment, or traditional agriculture. The other opposing cluster is large and combines actors which were part of clusters 1, 2, and 4 in period 1.

In period 3, scientists became less active in the supporter coalition, which was gradually taken over by other actor types. Business and media actors gradually left the opponent coalition and entered the supporter coalition. There was a clear change in the behavior of media actors in the different periods, probably reflecting the high level of media support for the government in China. Another possible explanation for the change in media behavior could involve the closer cooperation between the government and media aiming at increasing public biotechnological literacy (MARA, 2018). In any case, these results indicate that the Chinese government has attempted to influence public opinion on GM crops.

The social network cluster analysis for period 3 reveals four different communities, two belonging to the opponent coalition and the other two belonging to the supporter coalition. The small supporting cluster includes actors convinced that the opposition of others was due to a lack of knowledge concerning biotechnology, those trusting the safety of GMOs, and those supporting the placement of GM crops on the market as feed or food. The large supporting cluster combined all other reasons for support. In the small opposing cluster, many actors distrusted the government in general and worried about the transparency, assessment, and regulation of GM crops. They therefore opposed the promotion and cultivation of GM crops. The larger opposing cluster comprises actors who were former part of clusters 3 and 4 in period 1.

Interestingly, even after the policy support was announced in 2016, local governmental actors (e.g. from Heilongjiang and Qinghai) continued to post statements opposing GM crops. One explanation could be provincial government officials were eager to prevent unfavorable public opinion, largely out of concern for their career prospects (Chen et al., 2016). Statements from MARA appeared 1.5 times more often on Weibo than in the period before 2016. This indicates that the Chinese government was more active in its attempts to influence public opinion on GM crops in period 3.

In general, the results of the social network cluster analysis reveal the underlying conflict lines. It would be politically risky for the policy-makers to lift the blockage of GM crop cultivation in China at this stage, as the debate continued to be dominated by the opposition, albeit weakened. Under these circumstances, policy-makers apparently preferred to maintain policy stability instead of exposing themselves to the threat of losing regime legitimacy by changing the existing policy regime (Chen et al., 2016).

## 7. Conclusions and policy implications

Our analysis of Weibo posts between March 2013 and April 2020 reveals a public debate on GM crops that is characterized by strong public resistance against the commercialization of GM crops in China. Statements from opponents outnumbered those from supporters in all years. Consistent with previous studies, the smaller number of supporters might have been due to the availability of other channels (e.g. inside lobbying) that supporters of GM crops (e.g. seed companies) could use to influence decision-makers (Deng et al., 2019; Schaub and Metz, 2020). The analysis further shows how the government may have attempted to influence public opinion through active participation in the debate and by convincing other actors (e.g. the media) to change sides and join their coalition of support in the debate. Despite these efforts, public resistance to GMOs in China remains strong as shown by our results. The persistent dismissive public opinion on GMOs and the Chinese government’s concerns about political stability help to explain its hesitation to lift the current ban and allow additional cultivation of GM crops.

Our study provides scientific evidence to enhance systematic understanding of the public debate on Weibo concerning GM crops and the behavior of various stakeholders in China. Our analysis of Weibo posts reveals that the debate was characterized by two competing advocacy coalitions, regional differences, and changes in coalition membership over time. In general, the debate was dominated by relatively active opponents of GM crops, consisting largely of anonymous individuals. Their strategy involved eliciting emotions and disputing scientific evidence. In contrast, and consistent with findings from previous studies (Augoustinos et al., 2010; Mielby et al., 2013), we found that supporters primarily emphasized scientific evidence about food and environmental safety.

The structure of the debate might change in the coming years, as members of the government, business community, and media are becoming more involved in the debate, in a concerted effort to promote GM crops. This change started in late 2018, when MARA began its efforts to increase public knowledge about GM crops with the help of the media. Our results also reveal inconsistent behavior on the part of government representatives. Lower-level officials often oppose GM crops, in an attempt to be more in line with public opinion. Similar observations have been made by other scholars, who have attributed them to the desire to avoid ruining their own career prospects (Black et al., 2008; Chen et al., 2016).

The findings of our study have several policy implications. First, they could enhance the Chinese government’s understanding of the concerns of citizens and its ability to target these concerns more specifically when promoting the commercialization of GM crops, instead of merely repeating the potential benefits and safety of GM crops based on scientific evidence. One reason for opposition involves distrust in the government. This finding is consistent with a previous study indicating a lack of trust in government supervision in the area of food safety and labeling information (Liu et al., 2019), as well as GMOs in general (Zhang et al., 2010). Studies have demonstrated that trust is crucial in shaping public attitudes toward GMOs (Evenson and Santaniello, 2004; Kikulwe et al., 2011; Zhang et al., 2010). Regaining public trust is therefore vitally important to food policies relating to controversial topics (e.g. GMOs, food labeling), as well as other more general societal issues.

Our study indicates that the participation of companies in the public debate online is relatively low, but the mass media serve as an important channel through which the public can access information on GMOs. For companies with lobbying intentions, promoting GMOs through social media (e.g. Weibo) might therefore be a feasible supplement to direct governmental lobbying, given that MARA also considers socioeconomic and political factors when making decisions (USDA, 2015).

The information that we provide on the regional distribution and time dynamics of the public debate could help major players (e.g. companies with lobbying intentions) to adjust their strategies in time, thereby enhancing their ability to influence the public in specific regions in the future. Our study also contributes to the international food-policy debate, as its implications also apply to other countries aiming to advance the commercialization of GM crops or crops derived with genome editing, as they face strong public resistance in general. In some cases, public resistance could result in delays for science-based technologies. Learning from the public debate on GMOs in China could help improve policy design in the future, taking into account concerns from both the public and stakeholders. For example, before implementing a policy related to GMOs, the government could focus more on specific stakeholders or the public in a certain region by organizing workshops and forums for alleviating their concerns or regaining public trust. More knowledge transfer could be allocated to those target groups and target areas to bridge the gap between the science group and the public.

Although our study provides a promising starting point for analyzing the public debate on GM crops in China, the sample is limited to the most heated debate topics on Weibo over the past 7 years, as well as to actors who clearly expressed their attitudes toward GMOs. It is important to realize the potential difference between public opinion and public discussion based on the online platform. Further research on the GMO debate on Weibo is needed to test the robustness of our findings in a larger sample, but also on the use of social media platforms to influence public opinion.

Finally, one interesting, but perhaps not so surprising result is that when the government and the scientific community became more involved in the debate on Weibo with positive statements about GMOs, trust in the government increased. This might also have been the other way around, trust in the government increased resulting in more positive statements by scientists and the government. We find this argument less convincing. Nevertheless, one may ask if China is a special case or if the same could be expected for other countries or regions. This is in particular important for those countries and regions that have a strong interest in sustainable development. To realize this objective, using modern biotechnology could be extremely important (Purnhagen et al., 2021). Nevertheless, the use of modern biotechnology is viewed critically and in many cases opposed based on a wrong understanding of the facts (Fernbach et al., 2019). Governments might use social media to reduce opposition. If and how such a strategy might work outside China is still an open question.

## Supplemental Material

sj-docx-1-pus-10.1177_09636625211070150 – Supplemental material for Does China have a public debate on genetically modified organisms? A discourse network analysis of public debate on Weibo Click here for additional data file. Supplemental material, sj-docx-1-pus-10.1177_09636625211070150 for Does China have a public debate on genetically modified organisms? A discourse network analysis of public debate on Weibo by Yan Jin, Simon Schaub, Jale Tosun and Justus Wesseler in Public Understanding of Science

## Notes

1. We identify coalitions by coding whether actors agree or disagree with certain positions on GMOs, which does not necessarily lead to the identification of one GMO supporting and one GMO opposing coalition. For instance, some actors might hold positive views on some aspects of GMOs and negative views on other aspects, and thus form a third intermediary coalition.

2. Variation in activity in debates tends to create a bias where actors that are more active are more likely to reveal similar positions. This bias is corrected by dividing the sum of shared concepts by the sum of shared and nonshared concepts for each pair of actors (Leifeld, 2017).

3. The anonymous individuals are those Weibo users whose Weibo accounts are not verified and therefore, anonymous. We categorize them as the general public.

4. Given that the absolute spatial distance between two coalitions in Figure 1 is meaningless, a larger empty space between the two coalitions was removed for a clearer view. Please refer to Supplemental Appendix 3 for high-quality figures.

5. Given that the absolute spatial distance between two coalitions is meaningless, the empty space between the two coalitions was removed for a clearer view in Figures 2–4. Please refer to Supplemental Appendix 3 for high-quality figures.
