# Supplementary material for: Does China have a public debate on genetically modified organisms? A discourse network analysis of public debate on Weibo
Source: Public Underst Sci. 2022 Jan 27;31(6):732–50. doi: 10.1177/09636625211070150 (PMC9344491; doi:10.1177/09636625211070150)
Supplement: sj-docx-1-pus-10.1177_09636625211070150 – Supplemental material for Does China have a public debate on genetically modified organisms? A discourse network analysis of public debate on Weibo [file sj-docx-1-pus-10.1177_09636625211070150.docx]

**Does China Have a Public Debate on Genetically Modified Organisms? A Discourse Network Analysis of Public Debate on Weibo**

Yan Jin^1, 3^, Simon Schaub^2^, Jale Tosun^2^, and Justus Wesseler^1^

^1^Agricultural Economics and Rural Policy Group, Wageningen University, The Netherlands

^2^Institute of Political Science, Heidelberg Center for the Environment,

Heidelberg University, Germany

^3^Teagasc – Irish Agriculture and Food Development Authority, Ireland

Contents

[**Appendix 1. Weibo topics** 2](#_Toc90304741)

[**Appendix 2. Number of statements** 3](#_Toc90304742)

[**Appendix 3. Figures** 4](#_Toc90304743)

# **Appendix 1. Weibo topics**

Table A1. Weibo topics addressed in the study

| Topic | Topic | Number of contributions |
| --- | --- | --- |
| #不管几比几，不要转基因# | #No Matter What, No to GMOs# | 69,000+ |
| #拒绝转基因# | #Rejection to GMOs# | 54,000+ |
| #转基因食品# | #GM Food# | 25,000+ |
| #崔永元考察转基因# | #Cui Investigating GMOs# | 23,000+ |
| #转基因大米# | #GM Rice# (unprocessed) | 17,000+ |
| #转基因战争# | #GM War# | 8,433 |
| #农业转基因生物安全证书批准清单# | #Approval Lists of Biosafety Certificates# | 8,322 |
| #转基因滚出中国# | #GMOs Leave China# | 5,974 |
| #崔永元转基因# | #Cui and GMOs# | 5,603 |
| #转基因大豆# | #GM Soybeans# | 5,487 |
| #转基因作物# | #GM Crops# | 5,404 |
| #转基因大豆油# | #GM Soybean Oil# | 5,039 |
| #诺奖得主支持转基因# | #Nobel Prize Winners’ Supporting GMOs# | 2,089 |
| #转基因水稻# | #GM Rice# (processed) | 953 |

Note: Data accessed through April 15, 2020

# **Appendix 2. Number of statements**

Figure A1. Number of statements between May 2013 and April 2020

Note: The data represent the period between March 2013 and April 2020, due to the availability of data on Weibo. Data for 2013 and 2020 are therefore incomplete.

Source: Data collection from Weibo (2013–2020)

Table A2. Comparison of Mean Values for the Number of Statements on GMOs

| Two-sample *t*-test with unequal variances | | | | |
| --- | --- | --- | --- | --- |
| Groups | Mean | Standard Error | Observations | \|Difference\| |
| GMO opponents | 75.6 | 82.1 | 8 | 62.6 |
| GMO supporters | 13 | 7.7 | 8 |  |
| Pr(\|T\| > \|*t*\|) = 0.068, *t* = -2.15, Degrees of freedom = 7 | | | | |

# **Appendix 3. Figures**


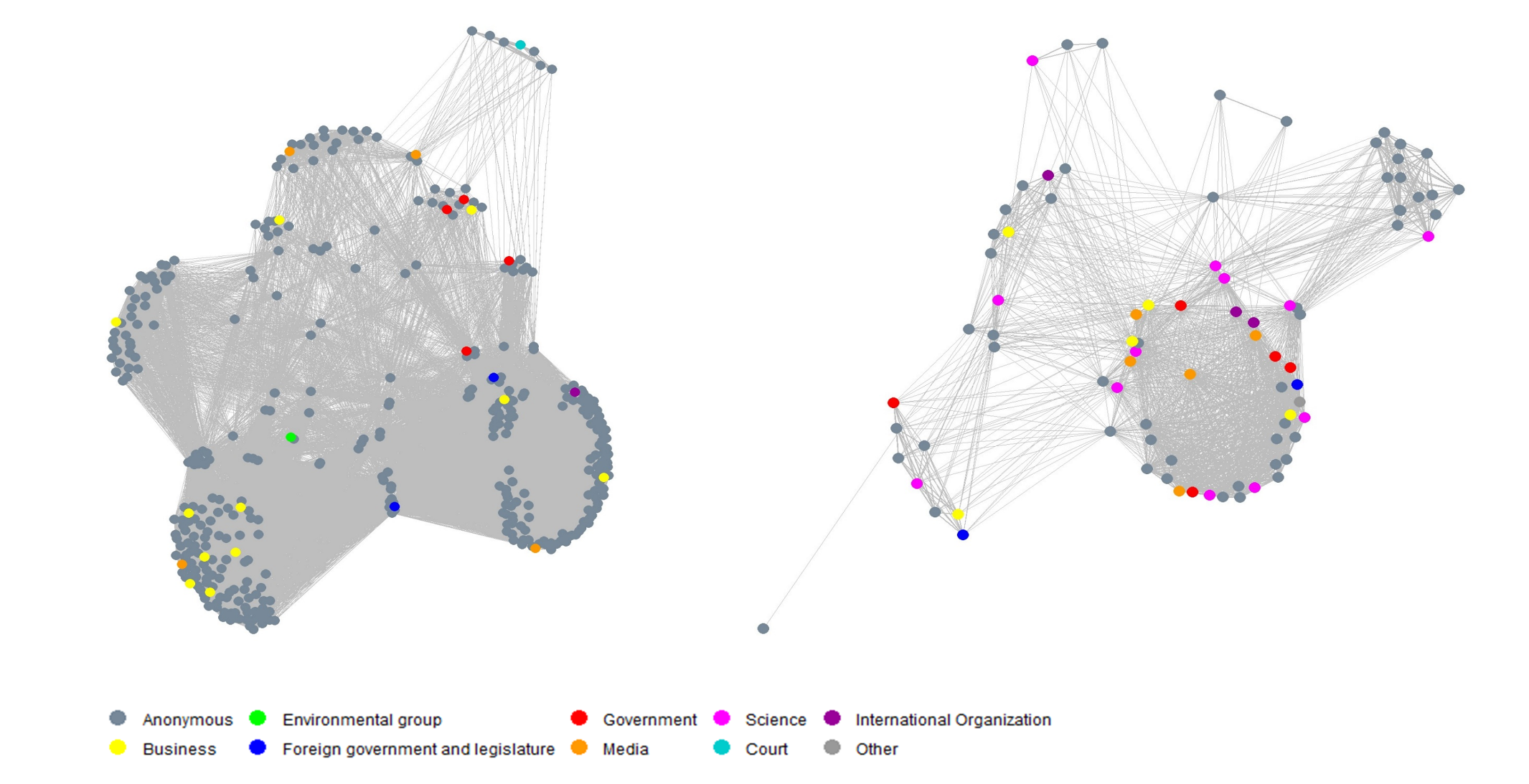


Figure 1. Visualization of the actor congruence network (left: opponents; right: supporters)


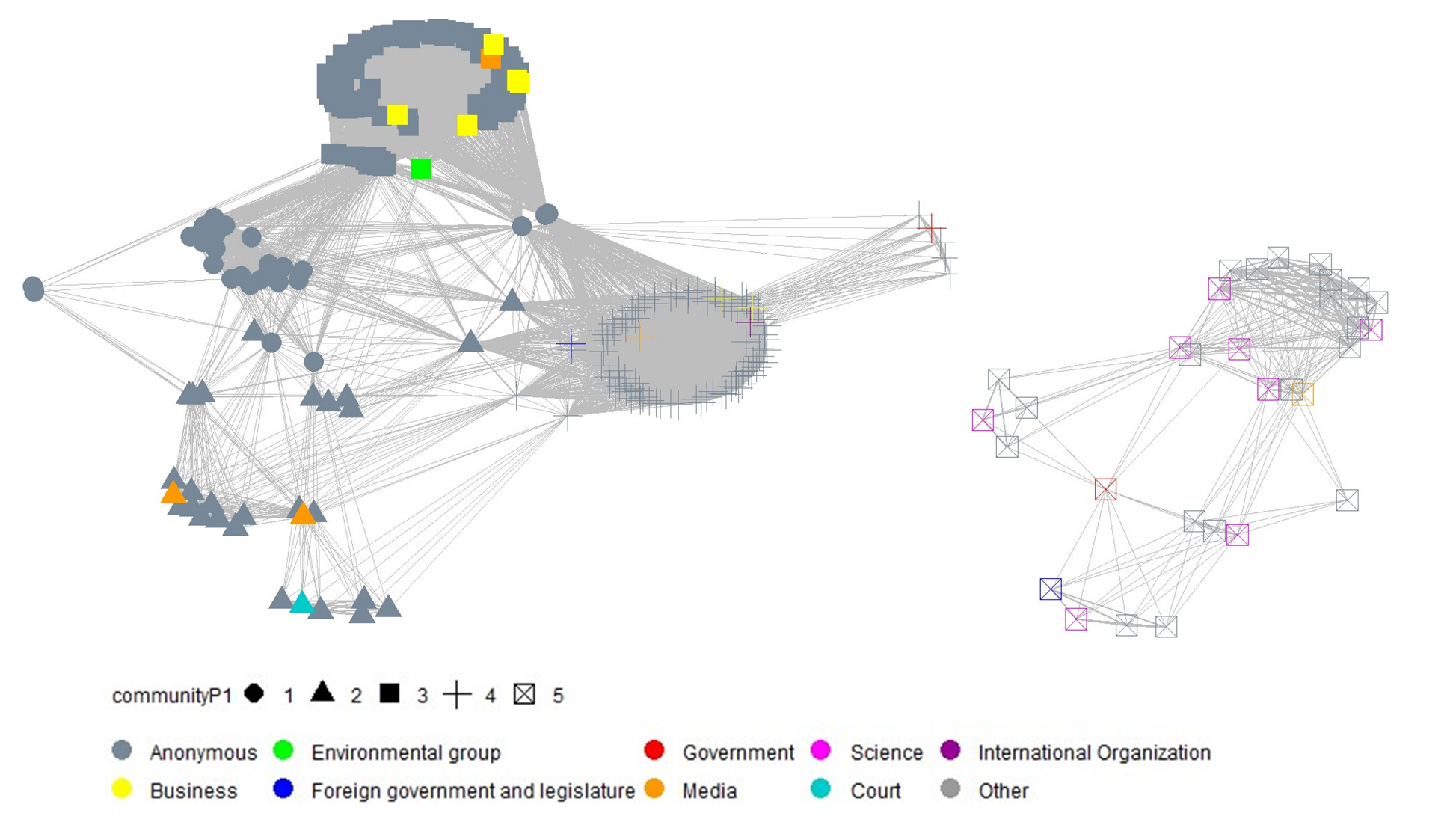


Figure 2. Visualization of time dynamics of the congruence network (left: opponents; right: supporters) and social network cluster analysis, based on the *Walktrap algorithm* (Period 1, 2013.05-2016.07, 352 statements)


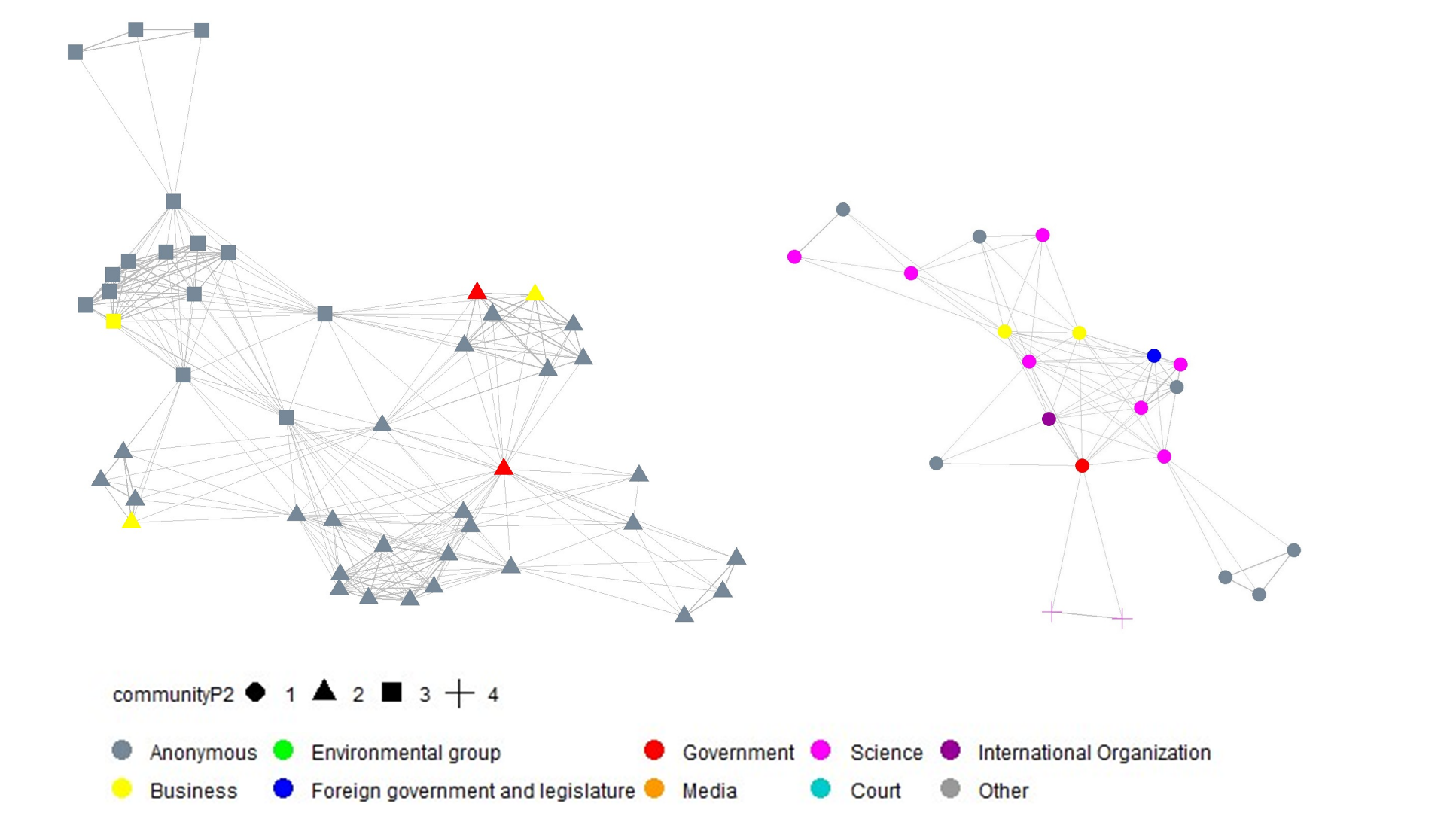


Figure 3. Visualization of time dynamics of the congruence network (left: opponents; right: supporters) and social network cluster analysis, based on the *Walktrap algorithm* (Period 2, 2016.08-2018.07, 104 statements)


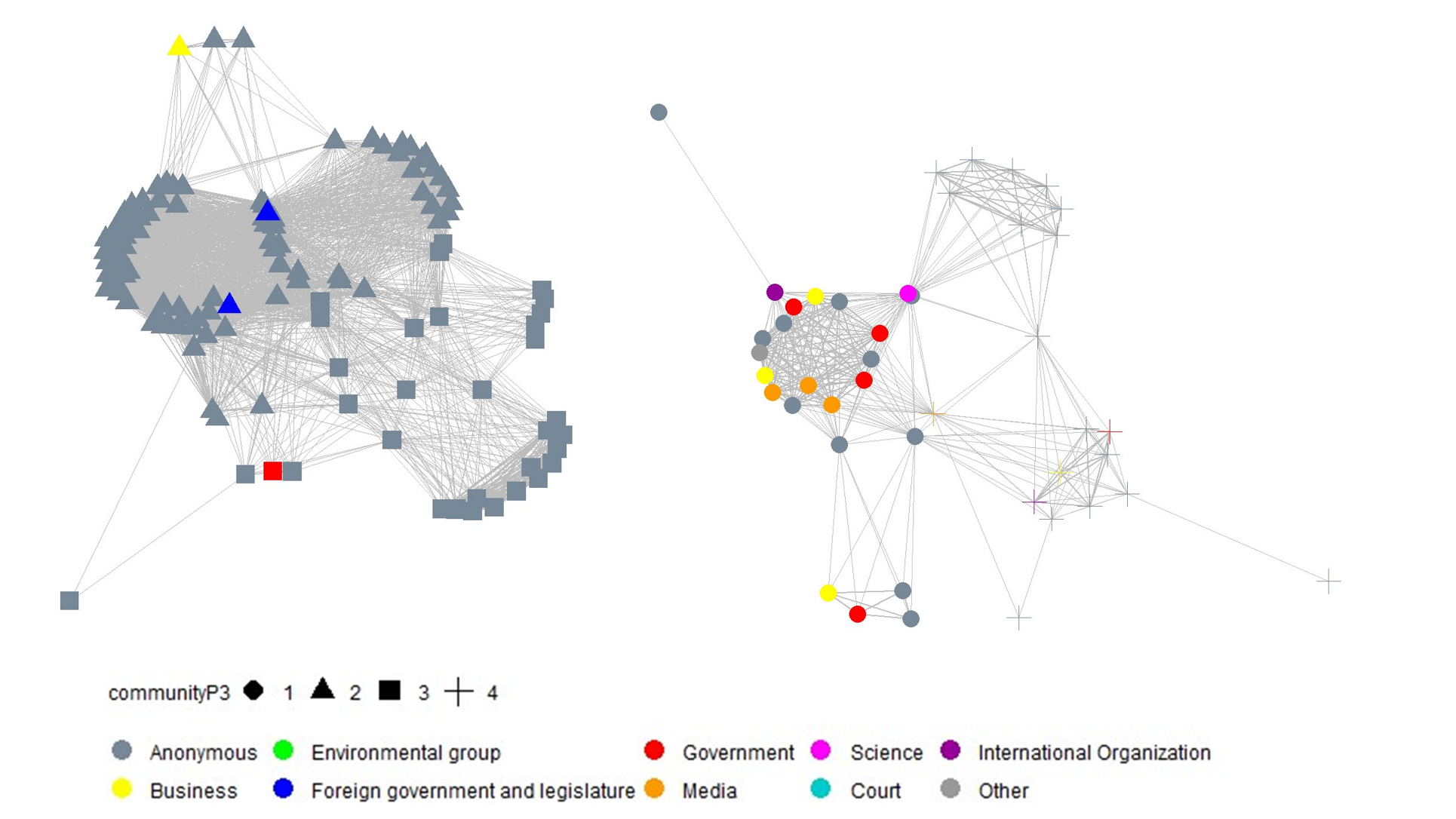


Figure 4. Visualization of time dynamics of the congruence network (left: opponents; right: supporters) and social network cluster analysis, based on the *Walktrap algorithm* (Period 3, 2018.08-2020.04, 322 statements)
